# A Guide to the Diseases of Children

**Published:** 1890-04

**Authors:** 


**A Guide to the Diseases of Children.** By JAMES FREDERICK GOODHART, M. D., F. R. C. P. Physician to Guy's Hospital, and Lecturer on Pathology in its Medical School, etc. Rearranged, revised, and edited by Louis Starr, M. D., Clinical Professor of Diseases of Children in the Hospital of the University of Pennsylvania; Physician to the Children's Hospital, Philadelphia, etc. Second American, from the Third English Edition with numerous formulæ and illustrations. 8vo, cloth, pp. viii., 772. Philadelphia: P. Blakiston, Son & Co. 1889. Buffalo: Peter Paul & Bro.

The first edition of this work was so well received and appreciated, that it only seems necessary to call attention to the appearance of the second edition, to insure for it a rapid demand. The American editor, Dr. Starr, has maintained in the present volume the valuable features of the former one, and has added some improvements which will increase its worth. He has rearranged it to some extent, left out the brackets indicating his additions, and added some illustrations. It is pleasant to find that the author appreciates the work of Dr. Starr, and has accepted some of his suggestions. There may be more exhaustive treatises on the subject of the Diseases of Children, but there is no better "guide," either for the student or busy physician, than the present volume. The book is presented in a pleasing form; the type, especially, is large and clear, the paper and binding excellent.

F. H. P.

---

**The Cure of Crooked and Otherwise Deformed Noses.** By JOHN B. ROBERTS, M. D. Professor of Anatomy and Surgery, in the Philadelphia Polyclinic; Lecturer on Anatomy in the University of Pennsylvania, etc. 8vo, cloth, pp. 24; Philadelphia: P. Blakiston, Son & Co. 1889.

This is simply a reprint of an address delivered before the Philadelphia County Medical Society, and published in the BUFFALO MEDICAL AND SURGICAL JOURNAL, for July, 1889. It is a good review of the subject from the standpoint of the general surgeon, though there are some things the specialist would take exception to. The punch and pin operation for instance, for the relief of septal deformity, is not nearly as good for the majority of cases as the saw operation of Bosworth. The article, however, as a whole, is judicious and comprehensive.

